# Tailoring glaucoma education using large language models: Addressing health disparities in patient comprehension

**DOI:** 10.1097/MD.0000000000041059

**Published:** 2025-01-10

**Authors:** Aidin C. Spina, Pirooz Fereydouni, Jordan N. Tang, Saman Andalib, Bryce G. Picton, Austin R. Fox

**Affiliations:** a School of Medicine, University of California, Irvine, Irvine, CA; b School of Medicine, Gavin Herbert Eye Institute at University of California, Irvine, Irvine, CA.

**Keywords:** ChatGPT, education, glaucoma, Large Language Model (LLM)

## Abstract

This study evaluates the efficacy of GPT-4, a Large Language Model, in simplifying medical literature for enhancing patient comprehension in glaucoma care. GPT-4 was used to transform published abstracts from 3 glaucoma journals (n = 62) and patient education materials (Patient Educational Model [PEMs], n = 9) to a 5th-grade reading level. GPT-4 was also prompted to generate de novo educational outputs at 6 different education levels (5th Grade, 8th Grade, High School, Associate’s, Bachelor’s and Doctorate). Readability of both transformed and de novo materials was quantified using Flesch Kincaid Grade Level (FKGL) and Flesch Reading Ease (FKRE) Score. Latent semantic analysis (LSA) using cosine similarity was applied to assess content consistency in transformed materials. The transformation of abstracts resulted in FKGL decreasing by an average of 3.21 points (30%, *P* < .001) and FKRE increasing by 28.6 points (66%, *P* < .001). For PEMs, FKGL decreased by 2.38 points (28%, *P* = .0272) and FKRE increased by 12.14 points (19%, *P* = .0459). LSA revealed high semantic consistency, with an average cosine similarity of 0.861 across all abstracts and 0.937 for PEMs, signifying topical themes were quantitatively shown to be consistent. This study shows that GPT-4 effectively simplifies medical information about glaucoma, making it more accessible while maintaining textual content. The improved readability scores for both transformed materials and GPT-4 generated content demonstrate its usefulness in patient education across different educational levels.

This research demonstrates that GPT-4 streamlines information on glaucoma, enhancing accessibility while preserving essential information. Enhanced readability scores for transformed materials and GPT-4 generated content validate its efficacy in patient education across diverse educational backgrounds.

## 1. Introduction

The advent of Large Language Models (LLMs) represents a transformative shift in the fields of Natural Language Processing, and machine learning.^[1]^ Among these, OpenAI’s (San Francisco) ChatGPT-4 stands out for its sophisticated approach to processing human language, enabling diverse applications such as translation, sentiment analysis, and text summarization.^[2,3]^ One notable use of LLMs is in simplifying medical texts. Often, medical literature utilizes complex terminology and sentence structures, making it difficult for the general public to comprehend.^[4]^ LLMs like ChatGPT-4 have the potential to democratize access to scientific advancements and medical knowledge, allowing the public to better understand their health conditions and the medical literature relevant to them.^[5,6]^

Glaucoma is a chronic and progressive eye disease requiring a multi-faceted approach to treatment, including medications, laser procedures, and surgeries.^[7]^ Patient adherence to these complex regimens is crucial for effective disease management and prevention of vision loss.^[8]^ Educating patients about the pathophysiology of glaucoma, the rationale behind its management as well as the importance of regular eye examinations is vital to improve compliance and patient outcomes.^[9]^ This education empowers patients to make informed decisions and actively participate in their treatment plan.^[9]^ LLMs have the potential to significantly enhance patient education and understanding by making information more accessible and tailored to individual needs.^[5,6]^ In clinical settings, they show potential to generate clear, personalized Patient Educational Models (PEMs), address specific concerns, and reinforce critical points from medical consultations.^[5,10]^

This study aims to evaluate the potential of GPT-4 in the context of glaucoma patient education. We hypothesize that LLMs like GPT-4 can effectively simplify complex medical information, enhancing patient understanding. Specifically, the study focuses on the ability of GPT-4 to transform research abstracts and synthesize new information tailored to individual patient needs in the field of glaucoma. The application of LLMs in this manner holds promise for improving patient understanding and compliance, ultimately contributing to better health outcomes.

## 2. Materials and methods

### 2.1. Transformation of published information: abstracts and PEMs

To elucidate the transformative capabilities of GPT-4, we first identified abstracts from recent research publications in 3 prominent glaucoma journals, including Ophthalmology Glaucoma (Volume 6, Issues 4 and 5, n = 23), Journal of Glaucoma (Volume 32, Issues 9 and 10, n = 24), and Journal of Current Glaucoma Practice (Volume 17, Issues 1 and 2, n = 14).^[11–16]^ Additionally, we examined publicly available PEMs available from the American Glaucoma Society (AGS).

The analysis of these published materials involved text assessment using Flesch Kincaid Grade Level (FKGL) and Reading Ease (FKRE) scores. The FKRE scale ranges from 0 to 100 with 100 representing a higher readability and 0 indicating a lower readability, while the FKGL scale ranges from 0 to 18 with 18 representing the most difficult text to comprehend.

FKGL and FKRE scores were calculated using the following formulae:^[17]^


$$\begin{array}{l} \begin{array}{lll} & FKRE=206.835-10.15\left(total\;words/total\;sentences\right) & -84.6\;(total\;syllables/total\;words)\; \end{array} \end{array}$$

$$\begin{array}{l} \begin{array}{lll} & FKGL=.39\left(total\;words/total\;sentences\right) & +11.8\;\left(total\;syllables/total\;words\right)-15.59\; \end{array} \end{array}$$

A Python (V3.11) script encoded with these formulas was utilized for all FKRE and FKGL quantifications.^[18]^

Subsequently, we employed GPT-4 to transform these abstracts to a 5th-grade reading level using the following prompt “Please rewrite this text to be readable at a 5th grade level. Do not include information not contained in the original text, and do not exclude information contained in the original text.”^[19]^ Following this transformation, another round of readability quantification was conducted to determine the readability of the published materials post-transformation.

### 2.2. Latent semantic analysis

Latent semantic analysis (LSA) is a technique used in natural language processing to analyze relationships between a set of documents and the terms and was applied to assess content consistency in transformed materials.^[20]^ Preprocessing, including stop word elimination and lemmatization, was performed to isolate relevant terms. Term Frequency-Inverse Document Frequency vectorization was done with max_features = 1000, max_df = .5, and min_df = 1. Pairwise LSA quantified the consistency of the information between the initial and altered texts as a cosine similarity value (0–1, 0 indicating no similarity and 1 indicating identical terms). Outlier analysis was subsequently performed to determine if any transformations had a statistically significant low LSA.^[20–22]^

### 2.3. De Novo outputs

Subsequently, to simulate how patients might use LLM technology, we prompted GPT-4 to generate “de novo” outputs. A standardized prompt regarding patient glaucoma management comprehension was iterated with output requests specified to match 6 different education levels: 5th grade (n = 5), 8th grade (n = 5), high school (n = 5), associate degree (n = 5), bachelor’s degree (n = 5) and doctorate degree (n = 5). The demographic information for the hypothetical patient was kept consistent across all prompts, with education level as the only variable that changed. The patient was described as a 47-year-old Iranian–American female, a small business owner residing in Orange, California. Outputs (n = 30) were assessed and scored for readability using FKRE and FKGL tests. Unidirectional ANOVA with Games-Howell post hoc analyses were performed to confirm the differences in the readability of the outputs for different education levels. IBM (Armonk) SPSS version 29.0.0.0 (241) was used for statistical analysis and GraphPad Prism (La Jolla) version 10.1.0 (264) was utilized for figure generation.

### 2.4. Online access and verified information sourcing

To further demonstrate GPT-4’s capability for synthesizing information from medically credible sources, we created a “Glaucoma Educator GPT” using OpenAI’s custom GPT tool. This functionality allows us to provide backend instructions to the model, which included the following:

“Your job is to create educational information for patients about their glaucoma diagnosis and prescribed interventions.

Perform the following tasks before generating your outputs:

1. Perform a BING search directly on the websites of the American Academy of Ophthalmology or the American Glaucoma Society. DIRECTLY CITE THIS INFORMATION IN YOUR OUTPUT.
2. Generate an output at a 5th grade reading level (THIS IS VERY IMPORTANT).
3. As you respond, cite each new “topic/heading” with a hyperlink referencing the source directly (ALWAYS DO THIS, EVERYTHING NEEDS A SOURCE).
4. ALWAYS generate the output at a FIFTH GRADE READING LEVEL!”

After optimizing these instructions, this Glaucoma Educator GPT can be provided with a prompt derived from our initial de novo prompts. This final output serves as a proof of concept, showcasing the model’s ability to adhere to given parameters for information retrieval from recognized, authoritative sources. Our Glaucoma Educator GPT is not available for public use and only a description of its output is provided in this manuscript to serve as an example of this technology's ability to source information from verified sources.

### 2.5. Ethics statement

Ethical approval was not necessary as the research did not involve human or animal subjects.

## 3. Results

### 3.1. Transformation of published information

#### 3.1.1. Abstracts

**Figure 1.**
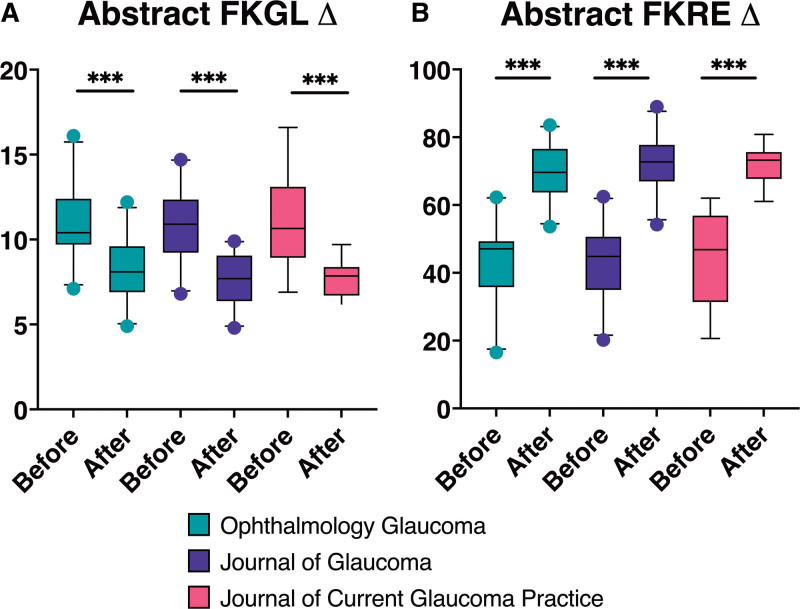
Transformation of abstracts by GPT-4. (A) Changes in FKGL scores of abstracts pre- and post- transformation, organized by journal (*P* < .001). (B) Changes in FKRE scores of abstracts pre- and post-transformation, organized by journal (*P* < .001). FKGL = Flesch Kincaid Grade Level, FKRE = Flesch Reading Ease.

In our comprehensive study of 62 abstracts, the transformation process led to significant readability improvements (Fig. 1A, B). Initially, the average FKGL of all published abstracts was 10.82 (SD 2.10), which decreased to 7.61 (SD 1.78) post-transformation, resulting in a significant reduction of 3.21 points (30%, *P* < .001, Fig. 1A). Similarly, the FKRE scores improved from an average of 43.64 (SD 11.85) pretransformation to 71.24 (SD 9.00) post-transformation, reflecting an increase of 28.60 points (66%, *P* < .001, Fig. 1B).

For abstracts published in the journal Ophthalmology Glaucoma, the pretransformation FKGL of 10.83 (SD 2.10) was reduced by 2.65 points (24.47% decrease, *P* < .001) to an average of 8.18 (SD 1.75) post-transformation (Fig. 1A). The FKRE scores rose from 43.19 (SD 11.75) to 69.88 (SD 8.57), an increase of 26.69 points (61.80% increase, *P* < .001, Fig. 1B). In the case of the Journal of Glaucoma, the FKGL scores decreased from 10.78 (SD 2.20) to 7.67 (SD 1.54), a reduction of 3.11 points (28.85% decrease, *P* < .001, Fig. 1A). The FKRE scores increased from 43.64 (SD 11.62) to 72.21 (SD 8.27), increasing by 28.56 points (65.47% increase, *P* < .001, Fig. 1B). For Current Glaucoma Practice, there was a reduction in FKGL from 10.89 (SD 2.59) to 7.69 (SD 1.17), equating to a decrease of 3.21 points (29.38% decrease, *P* < .001, Fig. 1A). The FKRE scores increased from 44.34 (SD 13.21) to 71.98 (SD 5.61), an increase of 27.63 points (62.34% increase, *P* < .001, Fig. 1B).

#### 3.1.2. Patient educational materials

**Figure 2.**
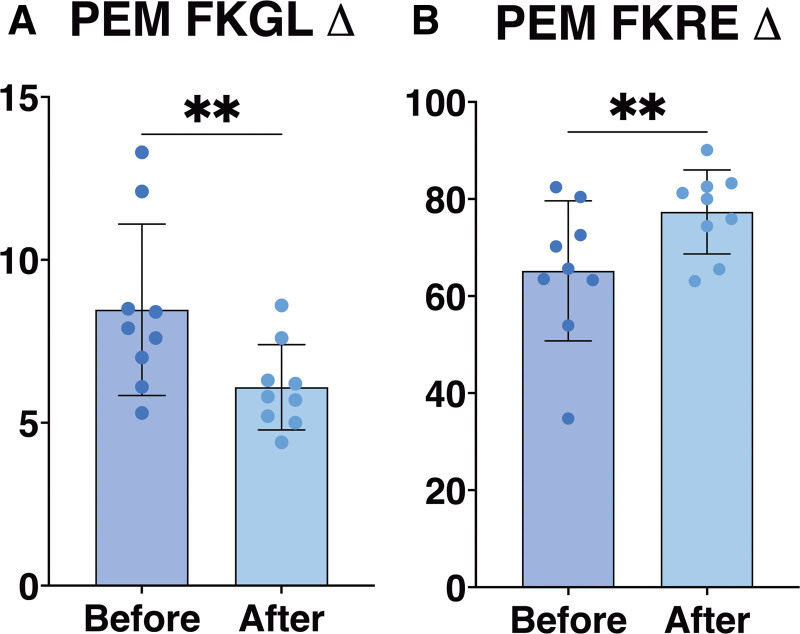
Transformation of PEMs by GPT-4. (A) Changes in FKGL scores for PEMs, pre- and post-transformation (*P* = .010). (B) Changes in FKRE scores for PEMs, pre- and post-transformation (*P* = .010). FKGL = Flesch Kincaid Grade Level, FKRE = Flesch Reading Ease, PEMs = Patient Educational Models.

In the analysis of the 9 PEMs, there was a decrease in FKGL from 8.47 to 6.09, and an increase in FKRE from 65.19 to 77.34 following content transformation (Fig. 2A, B). This equates to a reduction in FKGL by 2.38 points (28%, *P* = .0272) and an elevation in FKRE by 12.14 points (19%, *P* = .0459), with standard deviations narrowing from 2.63 to 1.31 for FKGL and from 14.44 to 8.66 for FKRE, reflecting a homogenization of readability post-transformation (Fig. 2A, B).

### 3.2. Latent semantic analysis

**Figure 3.**
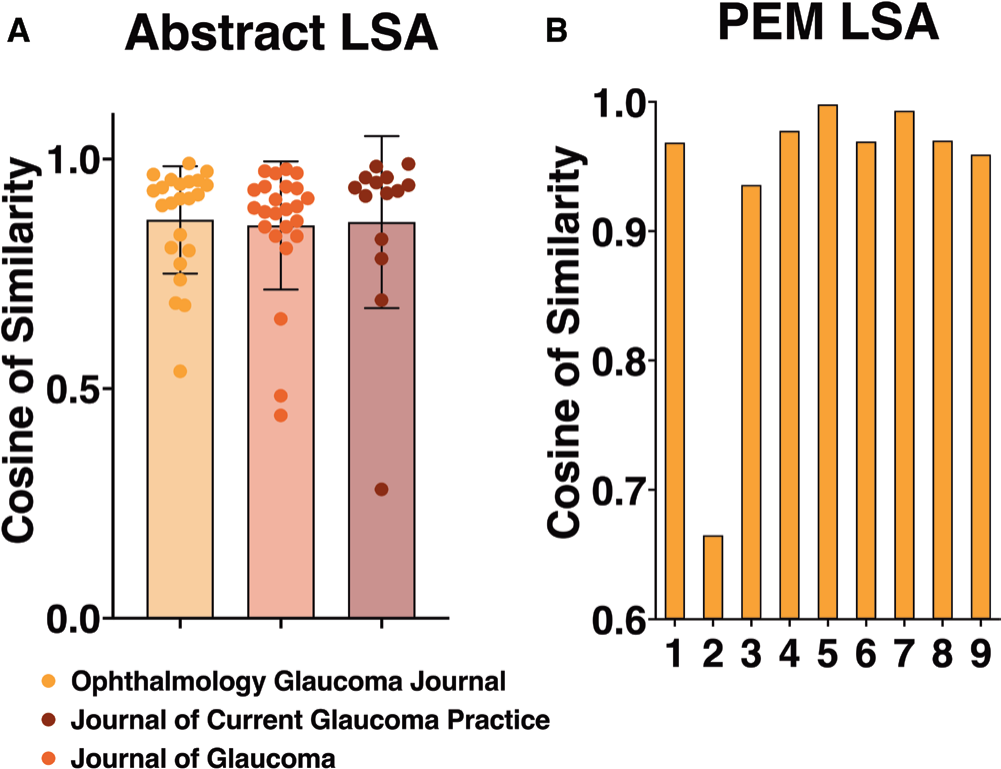
Latent semantic analysis of transformed abstracts and PEMs. (A) Cosine similarity of abstracts pre- and post-transformation. (B) Cosine similarity of PEMs pre- and post-transformation. PEMs = Patient Educational Models.

The average cosine of similarity for Ophthalmology Glaucoma was 0.868 (SD 0.117), for the Journal of Glaucoma 0.855 (SD 0.140), and for the Journal of Current Glaucoma Practice 0.863 (SD 0.187), indicating a general consistency in semantic content (Fig. 3A). The PEM demonstrated a higher mean cosine of similarity at 0.937 (SD 0.104), suggesting even greater consistency between raw and transformed materials (Fig. 3B).

In total, 70 abstracts and PEMs were analyzed, including 14 from the Journal of Current Glaucoma Practice, 24 from the Journal of Glaucoma, 23 from Ophthalmology Glaucoma, and 9 PEMs from the AGS. Our study identified 8 lower bound outliers across these materials, indicating significant deviations from common thematic content of the abstracts before and after transformation (Fig. 3A). These outliers were distributed as follows: 2 in the Journal of Current Glaucoma Practice (14.29%), 3 in the Journal of Glaucoma (12.5%), 2 in Ophthalmology Glaucoma (8.7%), and 1 in the PEMs (11.11%, Fig. 3A, B).

### 3.3. De Novo outputs

**Figure 4.**
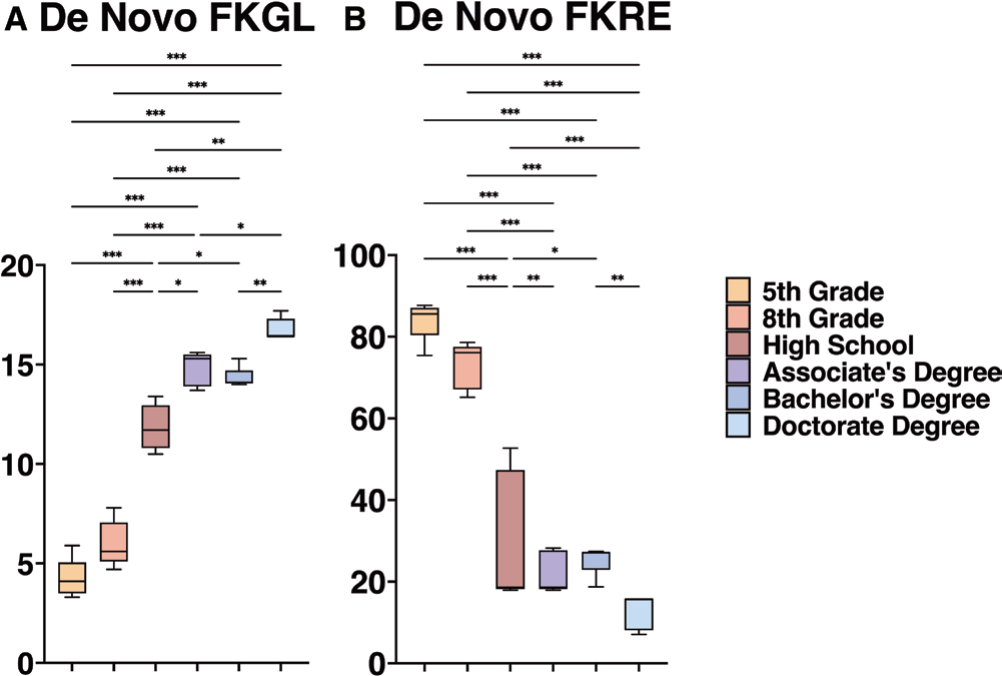
Readability scores for De Novo GPT-4 outputs. (A) FKGL scores for De Novo outputs (*** *P* < .001, ** *P* < .01, * *P* < .05). (B) FKRE scores for De Novo outputs (*** *P* < .001, ** *P* < .01, * *P* < .05). FKGL = Flesch Kincaid Grade Level, FKRE = Flesch Reading Ease.

In our study generating de novo outputs, we observed varying degrees of alignment between the readability levels of the outputs and the intended educational levels (Fig. 4A, B, Table 1). For 5th grade outputs, we found the average FKGL to be 4.24 with a standard deviation (SD) of 0.994, where 80% of the outputs aligned with the 5th-grade level (Fig. 4A). At the 8th grade level, the FKGL averaged 5.98 (SD 1.16), with each output readability falling within or below the 8th-grade reading level (Fig. 4A). For high school-level outputs, the average FKGL was 11.4 (SD 1.14), with 60% meeting the high school reading standard (Fig. 4A). Associate degree level outputs had a higher average FKGL of 14.82 (SD 0.858), however, none aligned with the expected associate level (Fig. 4A). Bachelor’s degree outputs presented an average FKGL of 14.32 (SD 0.550), with 100% aligning with this educational level (Fig. 4A). Doctorate degree outputs showed the highest FKGL at 16.76 (SD 0.568), with all outputs meeting the doctoral level (Fig. 4A).

**Table 1:** **Flesch Reading Ease Score interpretation based on the US grade level system.** ^[17]^

Score	School level (US)	Description
10–0	Professional	Extremely difficult to read. Only suitable university graduates
30–10	College graduate	Very difficult to read and comprehend
50–30	College	Difficult to read and comprehend
60–50	10th to 12th grade	Fairly difficult to read and comprehend
70–60	8th & 9th grade	“Plain English”
80–70	7th grade	Fairly easy to read and comprehend
90–80	6th grade	Easy to read and comprehend. Considered conversational English for speakers
100–90	5th grade	Extremely easy to read and comprehend

In the FKRE analysis, outputs for 8th grade, bachelor’s, and doctorate levels showed notable outcomes (Fig. 4B). The average FKRE score for 8th-grade outputs was 73.1 (SD 5.70), exceeding the targeted range of 70 to 60, thus indicating easier readability (Fig. 4B, Table 1). For the bachelor’s degree, the average FKRE was 25.5 (SD 3.78), well within the target range of 30 to 10 (Fig. 4B, Table 1). The doctorate degree outputs had an average FKRE of 12.7 (SD 4.26), surpassing the desired range of 10 to 0, also suggesting easier readability (Fig. 4B, Table 1). Conversely, for 5th grade, high school, and associate degree levels, the average FKRE scores were 84.12 (SD 4.96), 43.8 (SD 6.63), and 22.1 (SD 5.13) respectively (Fig. 4B, Table 1). These scores approached but did not consistently meet their target ranges of 100 to 90 for 5th grade, 60 to 50 for high school, and 50 to 30 for associate degree (Table 1). Notably, no individual outputs for 5th grade or associate degree were within the desired range, while only 20% of high school outputs achieved the FKRE target (Fig. 4B, Table 1).

ANOVA analysis demonstrated that as the educational level advanced from 5th grade to doctoral degrees, the FKGL scores consistently increased, indicating a rise in reading complexity (Fig. 4A). Conversely, the FKRE scores demonstrated a decrease, reflecting a corresponding decline in reading ease with each higher educational level (Fig. 4B). The Games-Howell post hoc analysis showed statistically significant differences in means for both FKRE and FKGL across almost all educational levels, except between 5th and 8th grades, and associate and bachelor’s degrees (*P* ≤ .05, Fig. 4A, B).

### 3.4. Online access and verified information sourcing

The output generated by our Glaucoma Educator GPT, when prompted to generate an output at a 5th grade reading level, had an FKGL of 6.70, and an FKRE of 73.27. The Glaucoma Educator GPT was able to source 3 unique “citations” all from verified sources 5 separate times throughout the output.^[23]^

## 4. Discussion

### 4.1. Transformation of published information: abstracts and PEMs

Analysis of the FKGL and FKRE scores, pre- and post-transformation using GPT-4, revealed a statistically significant enhancement in readability across the examined publications (Fig. 1A, B). The transformation of research abstracts from various glaucoma journals demonstrated comparable levels of success, suggesting that GPT-4 can effectively simplify content regardless of differences in structure, content, or reporting style. This uniformity in transformation underscores the potential of LLMs like GPT-4 in making current glaucoma research more accessible to patients.

However, the study also identified a limitation in the extent to which GPT-4 could simplify research abstracts. Despite specific instructions to reduce complexity to a 5th-grade reading level, the results showed that neither FKGL nor FKRE averages fully achieved this level of simplification (Fig. 1A, B). This shortfall is likely a consequence of the complex nature of original research publications, characterized by high FKGLs and low FKREs, necessitating a substantial reduction to meet the simplicity requested. Future investigations should focus on optimizing prompts to consistently achieve the desired readability levels. Moreover, advancements in LLM technology may soon enable more effective simplification with minimal prompting.

In contrast, the PEMs provided by the AGS demonstrated greater efficacy in achieving the intended simplification to a 5th-grade reading level (Fig. 2A, B). This enhanced level of success can be attributed, in part, to the initial lower reading level of these materials. This observation underscores an important principle in textual simplification: achieving the desired level of readability is more readily accomplished when the initial readability is already proximal to a lower target level.

The effectiveness of these transformations underscores a significant prospective application of LLMs in healthcare: democratizing access to the latest medical research. By simplifying intricate medical literature, LLMs have the potential to keep patients informed about the latest developments in their health conditions, such as glaucoma. This advancement is particularly impactful for patients with low health literacy (LHL), who have traditionally faced barriers in accessing such information.^[24,25]^ Similar research in other health-related topics, such as diabetes mellitus II, hypertension, and orthopedics, have shown positive early results, showcasing GLMs’ capability to improve readability and accessibility for patients with LHL.^[26,27]^ Furthermore, LLMs show potential beyond use on medical literature and patient materials. LLMs in 1 study were able to improve readability (FKRE and FKGL) in MRI radiology reports of varying complexity; in another study of MRI radiology reports, cohort of surgeons and radiologists surveyed on ChatGPT-simplified reports concluded the simplified reports were factually correct, reproducible, and did not present potential harm to patients.^[28,29]^

### 4.2. Latent semantic analysis

Our research findings indicate that GPT-4 can effectively transform most of the reviewed medical documents, preserving essential information (Fig. 3A, B). However, the presence of outliers within these transformations signals potential deviations from the source material. This highlights an area for further inquiry to identify and address such variances. This is particularly crucial in a medical context, where the precision of information is of utmost importance. Therefore, it is highly advisable for materials processed through LLMs such as GPT-4 for clinical purposes to undergo a review by an Ophthalmologist, ensuring their accuracy and appropriateness for educating patients.

The variation in standard deviations and the occurrence of lower bound outliers in our LSA of glaucoma-related journals underscore the complexity inherent in medical literature (Fig. 3A, B). This complexity is especially pronounced in the Journal of Current Glaucoma Practice, which showed a higher frequency of outliers, possibly due to its distinct thematic content or specialized terminology. Such insights highlight the crucial role of content analysis in the process of simplifying texts, aiming to retain the accuracy and continuity of information after transformation. In this scenario, LSA plays a pivotal role in ensuring the fidelity of content, monitoring for any inadvertent content loss or addition in the transformed texts.

Furthermore, while LLMs show promising capabilities in simplifying complex medical texts, consistent achievement of semantic similarity remains a challenge. The disparities in outlier frequencies and their impact on the integrity of the content indicate the need for continued development in LLM technology. As these models advance, we expect enhancements in their proficiency to not only simplify texts but also to preserve the accuracy of content. Such progress will likely bolster the utility of LLMs in patient education and in the broader field of medical communication.

### 4.3. De Novo outputs

In our study, we explored how LLMs like GPT-4 can be tailored to meet the diverse educational needs of patients. By varying patient educational backgrounds in our input prompts, we assessed GPT-4’s ability to adjust the complexity of its outputs. Our research demonstrates that GPT-4 can effectively generate responses across a broad spectrum of readability levels, from a 5th-grade reading level to a doctoral level. The findings demonstrate a clear trend whereas the educational level increases, the FKGL scores rise, and the FKRE scores decrease. This indicates that as the complexity of language increases with higher educational levels, the ease of reading correspondingly decreases. This inverse relationship between FKGL and FKRE scores is consistent across the different educational levels. ANOVA and post hoc analyses confirmed a consistently significant difference in the range of readability levels produced. The exceptions were between 5th and 8th grade, and associate vs bachelor’s degree levels, where the output readability differences, as measured by FKRE and FKGL, were not statistically significant. This high degree of adaptability highlights the potential of LLM technology as a versatile tool in patient education. As simplifying educational materials is often recommended, it’s important to recognize GPT-4’s capability to produce a wide range of comprehensible content for diverse educational backgrounds. Similar findings have been found in other research on GLMs’ ability to produce de novo information, for example when asked to generate information regarding atrial fibrillation and cardiac implantable devices, breast imaging, and rhinoplasty.^[30–32]^

Our analysis revealed that the readability of outputs, as measured by the FKGL, aligned more closely with our target readability levels compared to the FKRE scores. This discrepancy likely stems from the differing emphases of the 2 formulas: for instance, the FKRE formula places more weight on polysyllabic words than the FKGL. Although the FKRE scores did not match the expected education levels as precisely as the FKGL scores, both demonstrated a statistically significant stratification across the educational levels. This suggests that while FKRE scores did not align perfectly with the predefined levels, they still reflected a consistent pattern of readability adjustment. We recommend further investigation into text simplification methods to better understand how to structure prompts effectively. This could lead to more precisely tailored output readability, enhancing the utility of text for specific educational purposes.

### 4.4. Online access and verified information sourcing

Considering the study’s findings, GPT-4’s ability to generate clear and concise PEMs presents promising implications for future healthcare applications. Importantly, the example output demonstrated proficiency in sourcing information exclusively from the AAO and AGS, providing outputs with hyperlinks to the exact data points within these verified websites. This feature not only ensures the reliability of the information provided but also enhances the user experience by directing them to the verified information source, facilitating further learning and understanding. Such capabilities suggest that with appropriate physician guidance, patients may gain access to personalized and comprehensible healthcare information. The inclusion of hyperlinks to specific data points is particularly noteworthy, as it offers a transparent pathway for patients to explore their medical conditions in more depth. These findings support the potential of advanced LLMs to assist in disseminating accurate medical knowledge and simplifying complex information for patient education.

### 4.5. Limitations

This study acknowledges certain constraints that should be considered when interpreting the results. One of the primary limitations is the accessibility of GPT-4, the advanced LLM utilized in this research, which is not yet widely available for public use and currently costs $20 a month. Lastly, the influence of the constant demographic variables, including age, location, occupation and ethnicity included in all de novo input prompts on the outputs produced, remains uncertain While we hypothesize the influence of these demographic variables is negligible to the purposes of this study, they could serve as a potential area for future research.

Another major set of limitations, not directly related to the results of this study, are challenges associated with patient privacy and LLM use.^[33]^ There is a need for regulatory compliance and the challenges GLMs face in ensuring the privacy and security of sensitive user data, especially protected health information. The potential risks and legal complexities emphasize the importance of safeguarding patient privacy and complying with privacy laws in the deployment of these technologies in healthcare.^[34]^

## 5. Conclusion

GPT-4 demonstrates a notable capacity to distill complex medical literature and patient education content pertaining to glaucoma into more comprehensible formats. The implications of this proficiency are particularly profound for patients with LHL, who stand to benefit significantly from the enhanced accessibility of medical information. LSA confirms that the vast majority of transformed texts preserve the integrity of the original content, without introducing extraneous information. Furthermore, GPT-4’s capabilities extend to generating outputs across a spectrum of readability levels with considerable accuracy, as evidenced by FKGL assessments. Considering these capabilities, alongside GPT-4’s facility to reference and integrate verified information from online resources, this study delineates the extensive potential for leveraging LLM technology as an instructive resource for patients with glaucoma, enhancing their understanding and management of the condition.

## Acknowledgments

GPT-4 (ChatGPT, OpenAI) was utilized to generate the outputs that were analyzed in this manuscript as outlined in the methods section of this manuscript. Additionally, GPT-4 was utilized to provide feedback on grammar and syntax for drafts of this manuscript.

## Author contributions

**Conceptualization:** Aidin C. Spina, Saman Andalib, Austin R. Fox.

**Data curation:** Pirooz Fereydouni, Jordan N. Tang.

**Formal analysis:** Aidin C. Spina, Saman Andalib, Bryce G. Picton.

**Methodology:** Aidin C. Spina, Pirooz Fereydouni, Saman Andalib.

**Project administration:** Aidin C. Spina.

**Software:** Aidin C. Spina, Saman Andalib, Bryce G. Picton.

**Supervision:** Aidin C. Spina, Austin R. Fox.

**Validation:** Austin R. Fox.

**Writing – original draft:** Aidin C. Spina, Pirooz Fereydouni, Jordan N. Tang.

**Writing – review & editing:** Aidin C. Spina, Pirooz Fereydouni, Jordan N. Tang, Saman Andalib, Austin R. Fox.
